# Sensor Monitoring of Physical Activity to Improve Glucose Management in Diabetic Patients: A Review

**DOI:** 10.3390/s16040589

**Published:** 2016-04-23

**Authors:** Sandrine Ding, Michael Schumacher

**Affiliations:** 1 HESAV, University of Applied Sciences and Arts Western Switzerland (HES-SO), Av. Beaumont 21, Lausanne 1011, Switzerland; 2 Institute of Information Systems, University of Applied Sciences and Arts Western Switzerland (HES-SO), Techno-Pôle 3, Sierre 3960, Switzerland; michael.schumacher@hevs.ch

**Keywords:** diabetes, T1DM, exercise, sensor, physiological parameters, vital signs, blood glucose monitoring, ECG, accelerometer, algorithm

## Abstract

Diabetic individuals need to tightly control their blood glucose concentration. Several methods have been developed for this purpose, such as the finger-prick or continuous glucose monitoring systems (CGMs). However, these methods present the disadvantage of being invasive. Moreover, CGMs have limited accuracy, notably to detect hypoglycemia. It is also known that physical exercise, and even daily activity, disrupt glucose dynamics and can generate problems with blood glucose regulation during and after exercise. In order to deal with these challenges, devices for monitoring patients’ physical activity are currently under development. This review focuses on non-invasive sensors using physiological parameters related to physical exercise that were used to improve glucose monitoring in type 1 diabetes (T1DM) patients. These devices are promising for diabetes management. Indeed they permit to estimate glucose concentration either based solely on physical activity parameters or in conjunction with CGM or non-invasive CGM (NI-CGM) systems. In these last cases, the vital signals are used to modulate glucose estimations provided by the CGM and NI-CGM devices. Finally, this review indicates possible limitations of these new biosensors and outlines directions for future technologic developments.

## 1. Introduction

The careful control of glucose concentration is important for diabetic patients. This is particularly true for patients with type 1 diabetes mellitus (T1DM), where glucose monitoring reduces the risk of hypoglycemia, of cardiovascular disease with microvascular and macrovascular problems and of neurological abnormalities [1,2]. Controlling blood sugar also prevents patient death [2,3], which could result from loss of consciousness and heart failure. Glucose measurements are traditionally performed by skin punctures regularly throughout the day. However this method is invasive and results in pain to the patient. It is also well established that the finger-prick method affects patient compliance with glucose measurements [4].

Continuous glucose monitoring devices (CGMs) were developed to infer blood glucose levels in real-time, based on measurements of interstitial fluid glucose concentrations. They constitute point-of-care tests [5] that have enabled some improvements in the self-management of diabetes with reduced hypoglycemia and increased time spent in euglycemia [6,7,8]. However, these benefits are more evident in patients with poorly controlled diabetes who have recently used an insulin pump, in combination with the CGM [9]. More generally, the devices still present a number of limitations. First, the accuracy and reliability of CGMs are limited [3,10,11], notably during hypoglycemia [12]. As a consequence, the devices cannot fully replace the finger-stick monitoring but are better suited to complement it. Second, these systems involve the use of cannula inserted in the subcutaneous tissue of the abdomen and are accordingly still invasive.

A number of technologies have been proposed for noninvasively estimating blood glucose concentration. Termed “minimally invasive CGM” or “non-invasive continuous glucose monitoring” (NI-CGM), they were created with the hope of generating more regular, or even real-time, glucose measurements and thus allowing a more efficient self-monitoring. These technologies exploit the changes in the chemical and physical tissues properties caused by the presence of glucose molecules. These changes can be detected based on optical, chemical or electrical phenomena, for example via Raman spectroscopy, fluorescence technology [13], optical coherence tomography [14,15], optical polarimetry or reverse iontophoresis in the case of the Glucowatch Biographer [16]. These devices and their underlying technologies have been extensively reviewed in the literature (e.g., [15,17,18,19,20]). The accuracy of each of these technologies for estimating blood glucose is low [15,16,19] and challenges still lie ahead concerning the portability of the devices, their safety and their cost [15,19].

So far, NI-CGM technologies have generally only been used in very standardized conditions, such as experimental laboratories of hospitals (e.g., [14,21,22,23]). Yet, to be useful and widely usable, the devices must be precise and accurate in the conditions of daily living [20]. Accordingly, their measurements must be robust to sweating and variations in body temperature. These factors have often been cited as disruptive for non-invasive systems. The proper functioning of the device in everyday life and especially during periods of physical activity is essential because even a low physical activity after a meal affects significantly glucose variability in diabetic patients [24]. Moreover, hypoglycemia can occur during exercise, even moderate, or few hours later [25,26,27]. Being able to cope with these challenges is an important attribute of NI-CGM technologies, because participation in physical activity is recommended for T1DM without complications [28], resulting in both psychological and clinical benefits with improvements in cardiovascular function [29].

Currently, a number of biosensors, mostly non-invasive ones, have been developed for monitoring patients’ physical activity and cardiac data (e.g., electrocardiograms–ECGs). They have proved valuable for detecting the onset and the end of physical activity in T1DM patients. The combination of two sensors devices, an ActiGraph wGT3X-BT activity monitor (ActiGraph, Pensacola, FL, USA) and a heart rate monitor (Polar^®^ Electro Inc., Lake Success, NY, USA) can be used to detect the onset of physical activity within about five minutes by which time glucose levels measured by CGM is only ±1 mg/dL on average [30]. Similarly, a model applied to data from an accelerometer and heart rate monitor (Zephyr Biopatch, Zephyr Technology, Annapolis, MD, USA) worn by 13 T1DM inpatients have been shown to detect a moderate exercise with a good sensitivity (97.2%) and specificity (99.5%) [31].

Moreover recent studies used physical activity and vital sign sensors to maintain euglycemia and predict hypoglycemia. Some of these devices can directly estimate the blood glucose concentration based on physiological variables. The algorithms used to achieve this have been designed based on research demonstrating changes in cardiac characteristics, such as heart rate and QT interval, during hypoglycemia and hyperglycemia (e.g., [32,33,34,35,36,37,38,39]). Other devices integrate the parameters related to the physical activity and/or cardiac function in the estimation of blood glucose level provided by CGM or NI-CGM.

The aim of this article is review biosensors based on physiological parameters related to physical activity, that were used to improve glucose monitoring in T1DM patients. Section 2 provides beforehand an overview of biosensors used to analyze the influence of physical activity on glucose levels and the information they provide on glucose variations in T1DM individuals. Section 3 considers the estimation of glucose levels based solely on physiological signals, related to exercise, and provided by biosensors. Section 4 and Section 5 review the combined use of sensors monitoring physical activity and glucose tracking via CGMs and NI-CGMs, respectively. In each section the underlying designs of these technological advances are highlighted and their benefits and weaknesses discussed. Research studies evaluating these sensors on T1DM patients are detailed and assessed with regard to clinical outcomes (blood glucose level control) but, where data is available, also patient satisfaction. Based on the analysis of these emerging technologies we outline, in Section 6, directions for improvements in the design and performance of current and future devices.

## 2. Glucose Dynamic during Sensor-Monitored Physical Exercise

Predicting precise blood glucose concentration during active everyday life and physical exercise requires detailed information on the influence of physical activity on glucose level. To our knowledge, only two studies have addressed the precise relationship between exercise and glucose trends with biosensors, in T1DM patients. They performed a tight temporal tracking of vital signs during physical activity following a meal [24] and during moderate physical activity mimicking daily activities [40]. The technology used in both cases was the Physical Activity Monitoring System (PAMS; Table 1). This sensor combines two tri-axial accelerometers (CXL02LF3-R; Crossbow Technology, San Jose, CA, USA) and four inclinometers (CXTA02; Crossbow Technology) for determining body posture and the individual’s movements. Data were collected every half second. The system is quite cumbersome with the two accelerometers placed at the base of the spine and four inclinometers fastened on each side of the body at the trunk and thigh levels, and special underwear required for fitting them. Moreover, because the PAMS only provided data relating to physical activity, three devices were needed: the PAMS, the CGM and the insulin pump, if necessary for the patient.

In their research, Zecchin *et al.* [40] gathered data on control patients (N = 20) and T1DM patients (N = 19) over four days. For control and diabetic patients, the authors showed that moderate physical activity, corresponding to a daily activity, was associated with changes in glucose level, estimated by the first- and second-order glucose concentration time derivatives. Correlation degrees were stronger for diabetic patients but occurred 5–10 min later. For these individuals the decrease in glycemic concentration following exercise was maximal after 15 min. The increase in glucose level following the beginning of the rest period peaked after about 15 min. From a clinical point of view the slower glucose decline with physical activity should be taken into account by T1DM subjects when adjusting their basal insulin.

Physical activity was also found to significantly reduce postprandial glucose excursions in T1DM and healthy participants [24]. Thus, based on data collected in T1DM patients between 30 min before to 4.5 h after a meal, the excursions, estimated by the incremental glucose area above basal, was reduced by 59% (from 18.4 mmol/L/270 min for meals followed by inactivity to 7.5 mmol/L/270 min for meals followed by walking). For control participants, the decrease was 53% (from 9.6 mmol/L/270 min for meals followed by inactivity to 4.5 mmol/L/270 min for meals followed by walking). These results provide thorough quantitative evaluations of the effect of physical activity on glucose dynamic. For future research, this information should be added to the algorithm of closed-loop system for a better prediction of insulin infusion and a tighter control of glucose level when exercising.

## 3. Sensors Estimating Glucose Level Based on Physical Activity Signals

The glucose concentration can be directly estimated from physiological parameter values. This has been done using the multisensory device SenseWear^®^ Pro Armband (SWA [41]; Table 1). This multisensory technology was developed by the firm BodyMedia (Pittsburgh, PA, USA). As its name indicates, it is worn around the arm, and two ECG electrodes are placed on the arm and shoulder. The SenseWear^®^ collects physiological data from five types of in-built sensors. A two dimension accelerometer registers the position and movement of the arm and of the body, a heat-flux sensor and a thermistor measure the dissipated heat from the body, as well as the body and surrounding temperature, a galvanic sensor measures the conductivity of the skin, and two ECG electrodes measure the cardiac electrical activity.

In this system, the estimation of plasma glucose is based on data collected by the SWA that were downloaded through the CMS prerelease software version 1.0. The data collected from the biosensor were processed using a supervised machine learning based model. It integrates first an algorithm that defines the context (exercise *versus* no exercise) and then a regression model that provides glucose level estimates for the previously defined context. The quality of the model fit was assessed via a k-fold cross-validation in which all subjects minus one are used to train the model and the remaining subject validates the model. This procedure is repeated so that each subject is in turn used for validation.

The SenseWear^®^ device and model-based inference of plasma glucose levels was tested on patients with type 1 and type 2 diabetes [41]. Minute-by-minute estimates were produced by the armband. The study included 41 patients, of which 18 had T1DM. Patients were aged 18–65 years (mean 42.1 ± 13.8) and the majority were women. Patients with T1DM were diabetic since 10.0 ± 7.1 years. A CGM (iPro, Medtronic, Northridge, CA, USA) was placed on each of the patients. The system was not tested in activities of daily life but only in two experimental conditions. For the first of these, glucose levels were artificially raised while, for the second, patients had to walk for 60 min on a treadmill at a speed of approximately 4 km/h (2.5 mph). For both experiments, the glycemic values of T1DM patients estimated with the SWA were correlated with the reference values obtained directly from the glucose analyzer. The correlations were good for the first experiment (*r* = 0.70, (*p* < 0.05) and the second one ((*r* = 0.90, *p* < 0.05). These correlations were also similar to those obtained between values from the CGM and the reference values (experiment 1: (*r* = 0.75, *p* < 0.05; experiment 2: (*r* = 0.95, *p* < 0.05).

This study suggests that the sensor can provide reasonably good estimates of plasma glucose levels. Yet, both experiments also showed that the device is of limited use when it comes to alerting the patient of hypoglycemia, as hypoglycemic episodes occurred during both experiments. Compared to the reference values, the plasma glucose values estimated by SWA during these episodes in the first experiment were not clinically acceptable. Thus, 0% of the values were in area A + B according to Clarke error grid, although the SWA was switched on. In the experiment with the treadmill, 26% of the values were clinically acceptable (area A + B). These values should be compared to those obtained with CGM. Of the blood glucose levels measured by CGM during the hypoglycemic episodes occurring in experiments 1 and 2, respectively, 36% and 0% of the values fell into area A, and none in area B. Accordingly, the SWA and CGM did not provide accurate hypoglycemia predictions compared to the glucose meter.

In the experiments just described, glucose value estimates were obtained under standardized test conditions. The effectiveness of the SWA in T1D patients has yet to be assessed under conditions of everyday life and under uncontrolled physical activity. A SWA (SenseWear Pro3 Body Monitoring System) was tested under free-living conditions on one type 2 diabetic patient [53]. The authors used a mathematical model (Wiener model) to integrate the data provided by the sensor with other variables, such as those related to diet (e.g., carbohydrate and protein intake). The vast majority of estimates provided by the model were clinically acceptable with 90% of predicted glucose concentrations in area A and 7% in area B according to the Clarke error grid analysis. The percentages concerned all glycemic values (*i.e.*, euglycemia, hypo- and hyperglycemia) and not only hypoglycemia like the previous study [41]. In their article, Rollins *et al.* [53] also noted poorer model-based predictions of glucose levels in case of high hypoglycemia. The study, although involving only a single patient, showed an interesting approach to the estimation of blood glucose in the conditions of daily life.

One of the parameters included in this study [53] is energy expenditure. Yet, care should be taken with the energy expenditure (EE) estimation provided by the SenseWear Pro3. The EE estimation has been evaluated in older T1DM and T2DM individuals during sessions of walking on a treadmill at various speeds and inclines [54]. The EE values were shown to be weakly underestimated (by 8% ± 17%) when the subjects walked uphill (5 km/h and 5% incline) and strongly overestimated (by 81% ± 24%) in case of level walking (3 km/h with 0% incline) [55]. The large overestimation at low efforts, in particular, might cause problems when using activity data to infer blood glucose levels for diabetic patients with a poor physical condition.

Despite these drawbacks, the use of mobile sensors to assess blood glucose level directly through physical activity related parameters is very promising and should be further developed. Finally the only information given in these articles about patient satisfaction indicates that no pain associated with the use of the SWA was reported by the patients [41].

## 4. Integration of Sensors-Based Physiological Parameters with CGM Data

Estimates of plasma glucose with CGM are not always reliable and it has been shown that physical exercise disrupts blood glucose dynamics, and can render the maintenance of euglycemia more challenging [24,40]. As a consequence, some authors have developed an approach to integrate vital signals during exercise to increase the prediction accuracy of glucose levels, based on CGM data. Thus, the information on, for instance, the patient's physical activity or heart rate, is used not only to directly estimate glucose levels, as presented previously, but, in this case, to improve the accuracy of the algorithms involved in the development of artificial pancreas.

In this context, Stenerson *et al.* [42] used the Zephyr BioHarness^TM^ 3 of the Zephyr Technology Company (Annapolis, MD, USA; Table 1). It is worn as a belt at the chest level and combines two sensors. The acceleration data, estimated by a 3-axis accelerometer, and heart rate data are recorded every second. The patients need to wear a harness, but also a CGM and an insulin pump. In a first study, the authors [42] collected, in 22 T1DM patients, the physiological and life-style data issued from these three systems. Data were collected throughout the patients’ daily activities, on average during 4.9 days (from 1 to 16 days). Glucose levels estimated by the CGM were obtained every five minutes. The authors developed an improved algorithm predicting glucose levels and stopping the insulin pump when the level is below the threshold of 70 mg/dL. With simulations, the authors pointed out that if the original algorithm reduces the number of hypoglycemia incidents by 62%, adding information on heart rate (HR) allows to reduce their frequency by 71% and data on acceleration by 74%. The combination of data of HR and acceleration reduces hypoglycemia by 76%. These results show the benefit of accelerometer data but do not justify the use of a heart rate sensor. However, data on the number of false positives hypoglycemia (*i.e.*, hypoglycemia detected by simulation but not real) were not mentioned.

The research by simulation was completed by a study of 18 T1DM patients gathered for a football game [43]. This study was designed to test the effectiveness of the improved algorithm with the acceleration data. The results however indicated that the algorithm has failed to prevent hypoglycemia in patients using it compared to patients using their usual basal insulin rate. Patients on algorithmic glycemic control did not show higher rates of hyperglycemia. This is an advantage since the current practice is to turn off the insulin pump before exercise, which often leads to hyperglycemia. In terms of telemedicine, in this study on Zephyr, the data from the accelerometer can be read in real time through the SenseView application (Mobili d.o.o., Ljubljana, Slovenia) on a mobile phone. It remains however that this system is only at an experimental stage since the insulin pump of patients using the algorithm was activated and deactivated manually by the staff in charge of the study. Finally, no adverse effect has been shown due to the wearing the Zephyr system.

If Stenerson *et al.* [42,43] retained the acceleration as the most important signal to integrate in the algorithm, in other studies, information on HR was included in order to modulate the amount of injected insulin [44,56,57]. Heart rate data were collected using a RS800CX sports watch from the Polar^®^ firm (Lake Success, NY, USA; Table 1). These data were integrated into an algorithm implemented on a DiAs artificial pancreas platform. DiAs was also connected to an insulin pump and to a CGM. It runs on an Android^®^ phone (Google, Mountain View, CA, USA) and is controlled by the patient that may introduce information such as the carbohydrate intake.

To evaluate this approach, the authors performed a study on 12 T1DM patients with a randomized crossover design [44]. They compared the effectiveness of the classical algorithm with the one enriched with heart rate values to prevent hypoglycemia during exercise. They showed a significant decrease of the reduction of blood glucose during exercise, and reduced hypoglycemia during moderate exercise, although this last difference was not significant. The advantages of this system, during the recovery phase and during the night, are less clear. Finally in case of physical activity, the system DiAs is manually informed by pressing a button when the HR exceeds 125% of the patient’s HR at rest. It should be further optimized to become automatic and also controlled to limit false alerts.

Other authors focused also on the heart rate signals, but using different devices. Thus, heart rate variability (HRV) was collected through lead II with a digital Holter monitor (SpiderView Plus, ELA Medical, Montrouge, France; Table 1) and combined with glucose values measured with CGM to predict and to improve the detection of hypoglycemia [45,46]. The combination initially tested in a clinical research setting [45] was evaluated in free-living conditions on 21 adult patients [46]. The algorithm combining glucose values with those of HRV shows a good response for both the sensitivity and specificity of hypoglycemia detection with, respectively, values of 91% and 100% for 20 min prediction interval.

The SenseWear, employed by Sobel *et al.* [41] to directly estimate glucose concentration (see Section 3), has also been used for combining physiological variables with blood glucose measurements obtained by CGM [47,48]. This combination is expected to provide better estimates of the insulin amount to inject. In these studies the major physiological parameters taken into account to indicate physical activity are the energy expenditure and the galvanic skin response. With the addition of these physiological data, glucose levels are better controlled. This adjustment is carried out directly, *i.e.*, without a user intervention to announce, to the system, the patient’s physical activity or food intake [47]. The device has been enriched by an alarm indicating efficiently the risk of hypoglycemia before their occurrence [48].

As mentioned, these studies did not directly estimate glucose levels from the sensor-based physiological values but rather combine these data with CGM measurements to provide an increased precision in glucose concentration predictions. This approach offers an interesting reflection on ways to combine, with mathematical algorithms, plasma glucose data with physical activity levels. They also allow an assessment of various sensors (Zephyr Bioharness, Sports Watch…) that could be used in other settings.

## 5. Integration of Sensors-Based Physiological Parameters with NI-CGM

In the last section we pointed out that physiological data can be combined with glucose level values measured by CGM. Similarly research has been conducted to improve the accuracy of NI-CGMs through the integration of several physiological factors [23,49,50]. These studies were conducted with the Multisensor Glucose Monitoring System (MGMS) developed by Solianis Monitoring AG (Zurich, Switzerland; Table 1). The device includes dielectric spectroscopy and optical sensors. Glucose level variations engenders changes in the properties of tissues, notably in cell membrane conductivity, that are tracked by the dielectric spectroscopy electrodes. An important feature is that the sensors for estimating glucose concentration as well as those monitoring potential disturbing physiological factors are integrated in the same device. Thereby, the apparatus, worn on the upper arm, also tracks the subject’s temperature, sweating and movement, among others. The monitoring of glucose levels changes through this system was subsequently improved through the implementation of more sophisticated mathematical models combining the various variables monitored by the device [49,50].

The results obtained are globally good, with 92% of the results in the A+B region of the Clarke error grid analysis [50]. Nevertheless, it would have been interesting to know if the behavior of the system is specifically reliable under hypoglycemia and hyperglycemia since these deviations in glucose levels were provoked in the patients.

One study has used Monte Carlo simulations to test the robustness of the system to a disruptive factor, sweating [51]. The authors showed that the system does not need to be re-calibrated after sweating event, which is appealing for use in everyday life.

The SensiumVitals (Sensium Healthcare Ltd, London, UK) is another device that combines multiple sensors into a small and lightweight single-use system. The device can measure various physiological parameters, including heart and respiratory rates, physical activity, blood pH or glucose level [58,59]. Blood glucose is monitored through an ion sensitive field effect transistor. The principle is based on variations in ion concentration resulting from glucose level changes [60]. The values of the physiological variables can be transmitted wirelessly. The reliability of this system for the estimation of cardiac and respiratory rates were evaluated with diabetic inpatients [48]. The SensiumVitals accuracy assessment does not appear to have been conducted for glucose level estimation on inpatients or outpatients. The integration of different physiological parameters in the determination of glucose level has not been approached either.

## 6. Conclusions 

The reviewed studies indicate the appeal of devices using physiological signals related to physical activity and their potential to improve the management of diabetes in a near future. Thus physical activity, although beneficial for diabetic patients, constitutes an element that disrupts glucose dynamics and generates blood glucose regulation problems during and after exercise. These sensors provided valuable results to estimate glucose concentration either based solely on physical activity parameters or in conjunction with CGM or NI-CGM systems. In these last cases, vital signs are used to modulate the glucose estimations provided by the CGM and NI-CGM devices.

These systems tracking physiological signals were assessed for their validity and accuracy to infer blood levels and to detect hypoglycemia. In this respect, their performance was often compared with CGM-based glucose estimations. Nevertheless, evaluations of their therapeutic superiority compared to gold standard treatment globally still need to be conducted. This would for instance allow one to determine if the new devices reduce the risk of hypoglycemia or if the glycaemia is adequately controlled in the long-term. The use of the largely recognized glycated hemoglobin (HbA1c) would allow to appreciate their efficiency to control blood glucose for three months [61].

In terms of communication, a key point concerns the need to develop compatible systems with the computerized patient records of clinical institutions [62,63]. This will promote the sharing of patient data between healthcare providers (doctors, nurses...) and should improve patient care [64,65]. The interoperability of multisensory devices for diabetic patients has been investigated to allow their connection with health monitoring platforms [66,67]. The proposed architecture follow the standards of Health Level 7 (HL7). Interoperability between systems is essential to ensure changes in the functioning of care. Healthcare provision initially mostly occurring in hospital settings and private practice, moves to a patient-centered process, much more open as occurring at all times and in all places [68,69]. Data could also be combined with other sources of information such as "social media" to generate clinical and well-being recommendations [70].

Wireless data transmission, to health professionals, to the patient and to his relatives, is not yet possible with all devices. Information and communication technology (ICT) will nevertheless undoubtedly bring an additional advantage to biosensors and their users. ICT offers to diabetic individuals a diversity of solutions to help them better manage the disease. ICT can for instance encompass internet, mobile phone applications (mHealth: [71,72,73]), and telemedicine technologies based on decision support. These technologies were, and still are, the subject of much literature reviews showing a general, although mild, advantage of these technologies for diabetic patients [74,75,76,77]. Notably they facilitate patient self-management [63], a better control of nutrition [76] and a metabolic control pointed out by a low reduction in glycated hemoglobin [78]. The patients also appear satisfied with these systems and present enhanced well-being and feeling of disease control [79].

Patient satisfaction has not been investigated for the presented devices tracking physical activity parameters. Only sparse general information on patients was mentioned in the articles, such as the absence of pain or discomfort. Studying patient satisfaction in regard with new technologies is in fact not a generality. A literature review indicates that this outcome is envisaged in less than 50% of the studies testing ICT [63]. Yet it is well known that patient satisfaction constitutes a significant part for a successful implementation. However, in recent years, primary studies published on patient satisfaction with technological advances have increased (e.g., [80,81]), as well as systematic reviews (e.g., [82]).

## Figures and Tables

**Table 1:** Sensors tested in relation with physical activity in T1DM patients and their specific use in research articles.

General Purpose	Product	Company	Sensors	Specific Use in the Articles
Monitoring glucose dynamic during physical exercise	Physical Activity Monitoring System (PAMS)	Crossbow Technology, San Jose, CA, USA	-2 tri-axial accelerometers (CXL02LF3-R)	Evaluation of glucose dynamic during physical exercise [24,40]
			-4 inclinometers (CXTA02)	
Physiological signals to estimate glucose level	BodyMedia SenseWear^®^ Pro Armband	SWA; BodyMedia, Inc, Pittsburgh, PA, USA	-A 2-axis accelerometer	Direct estimation of glucose level based on multisensor data [41]
			-Heat-flux sensor	
			-Thermistors	
			-Galvanic skin response sensor	
			-ECG electrodes	
Vital signals and CGM	Zephyr BioHarness^TM^ 3	Zephyr Technology, Annapolis, MD, USA	-Heart rate -A 3-axis accelerometer	Integration of heart rate and accelerometer monitoring in the glucose level estimation algorithm [42]
				Integration of accelerometer monitoring in the glucose level estimation algorithm [43]
	Sport Watch: Polar: model RS800CX	Polar^®^, Lake Success, NY, USA	-Heart rate	Integration of heart rate monitoring in the glucose level estimation algorithm [44]
	Digital Holter monitor, SpiderView Plus	ELA Medical, Montrouge, France	-ECG monitor	Integration of heart rate variability in the glucose level estimation algorithm [45,46]
	BodyMedia SenseWear^®^ Pro3 Armband	SWA; BodyMedia, Inc, Pittsburgh, PA, USA	-A 2-axis accelerometer	Integration of energy expenditure and galvanic skin response in a glucose level estimation algorithm [47,48]
			-Heat-flux sensor	
			-Thermistors	
			-Galvanic skin response sensor	
			-ECG electrodes	
Physical activity and NI-CGM	Multisensor Glucose Monitoring System (MGMS)	Solianis Monitoring AG , Zurich, Switzerland	-Accelerometer	Integration of temperature, sweat and acceleration and position in the glucose level estimation algorithm [23,49,50,51]
			-Temperature sensor	
			-Humidity sensor	
			-Optical sensor	
			-Dielectric spectroscopy (for glucose monitoring)	
	SensiumVitals	Sensium Healthcare Ltd, London, UK	-Heart rate	Reliability of the cardiac and respiratory rates estimates [52]
			-Respiratory rate	
			-Physical activity	
			-Blood pH	
			-Glucose level	

## Abbreviations

The following abbreviations are used in this manuscript:

CGM: Continuous Glucose Monitoring
ECG: Electrocardiogram
HbA1c: Glycated hemoglobin
HL7: Health Level 7
HR: Heart Rate
HRV: Heart Rate Variability
ICT: Information and Communication Technology
MGMS: Multisensor Glucose Monitoring System
NI-CGM: Non-Invasive Continuous Glucose Monitoring
PAMS: Physical Activity Monitoring System
SWA: SenseWear^®^ Pro Armband
T1DM: Type 1 diabetes mellitus
